# Screening for cervical cancer: when theory meets reality

**DOI:** 10.1186/1471-2407-11-240

**Published:** 2011-06-13

**Authors:** Mari Nygård

**Affiliations:** 1 Fr. Nansens vei 19, P.O. Box 5313 Majorstuen, N-0304, Oslo, Norway

## Abstract

Cervical cancer screening reduces morbidity and mortality due to cervical cancer. However, there are many factors that determine the success of any cervical cancer prevention effort: the prevalence of human papillomavirus infection in general population, the existence of an organized screening program and the corresponding coverage, the existence and quality of the field and laboratory facilities for screening and diagnostic follow-up, and the facilities available for treating diagnosed lesions. Monitoring the patient path or "chain of action" for each patient with an abnormal screening result is of crucial importance. Cost-effectiveness models are widely used by decision-makers to determine which cervical cancer screening program would maximize health benefits within a given, usually limited, set of resources. Regardless of their level of sophistication, however, these models cannot replace empirical evaluations of the effectiveness of screening programs.

Cervical cancer prevention activities need to be monitored and evaluated in each country where they are introduced to see that they meet performance standards. Policy-makers responsible for allocating resources for cervical cancer prevention have a duty to allocate resources not only for cervical cancer screening, but also for screening program surveillance.

## Introduction

In the medical field, disease prevention is often considered a cost-effective alternative to treatment. This statement is especially true for cervical cancer, where late-stage treatment is expensive and the outcome generally poor. Indeed, in Norway the 5-year relative survival rate for patients with late-stage cancer at the time of diagnosis has remained largely unchanged since 1956, hovering around 10%. In contrast, the same figure is over 90% for patients with stage I cancer [1]. Through screening individuals with asymptomatic preinvasive lesions are identified and treated to halt the process of cancer development. These findings imply that early diagnosis and treatment of cervical disease comprise a powerful strategy to combat the morbidity and mortality associated with cervical cancer. Unfortunately, implementation of these strategies in some parts of the world is not always feasible, and recently the International Agency for Research on Cancer reported that cervical cancer is still the second most common cancer worldwide, and disproportionately affects low-to-medium-income countries [2].

There is no doubt that cervical cancer screening reduces the morbidity and mortality due to cervical cancer. In order to determine which screening model would maximize health benefits within a given set of limited resources, decision makers often use cost-effectiveness models. During the last decade, results from mathematical modeling studies have become increasingly important in policy-making discussions on whether to implement human papillomavirus (HPV) vaccination and/or cervical cancer screening, as well as in the discussions of different screening tests and regimens [3]. Mathematical modeling is a powerful tool allowing the comparison of different screening regimens without performing an empirical study.

In the current issue, Shi et al. report on the cost-effectiveness of various cervical cancer screening strategies using an advanced mathematical model based on the natural history of cervical cancer. The model was specifically adapted to the Chinese context and applied different screening algorithms that are applicable to health care systems in rural China. Based on their model, the authors concluded that primary screening with a new molecular test, *care*HPV performs better than visual inspection methods in rural China, particularly if it is used as part of an orgnized screening programs.

In the current commentary, various aspects of cervical cancer screening will be discussed, based on the experiences of the Nordic countries.

## Discussion

The concept of cervical cancer screening is not new and dates back to the 1940s. The identification of preinvasive cervical lesions is possible through a combination of Pap smear and histological verification, and allows for appropriate treatment, i.e., removal or destruction of preinvasive lesions, which interrupts the natural course of cervical cancer and stops disease progression. The challenge lies, however, in making screening and treatment available to all women at risk.

**Figure 1:**
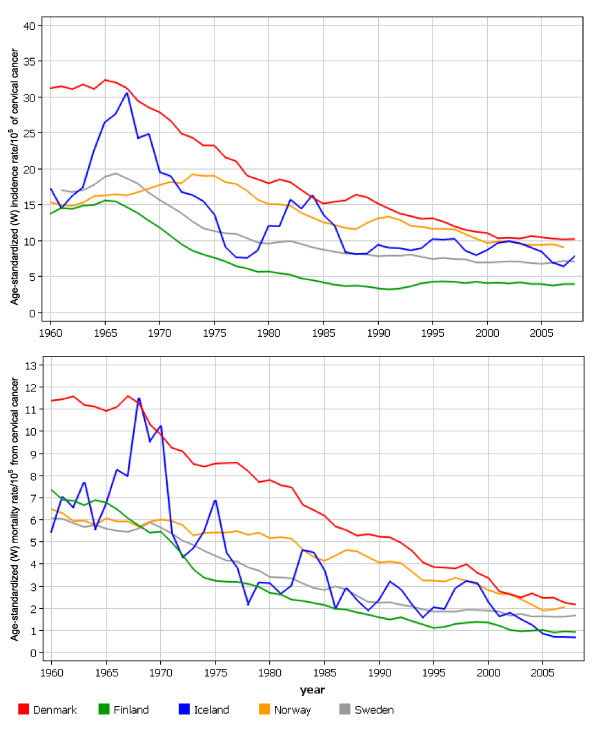
**Age-standarized (world standard population) incidence of (upper graf) and mortality (lower graph) from cervical cancer/10^5 ^in Denmark, Finland, Iceland, Norway and Sweden in 1945-2008**.

Countries that have implemented cervical cancer screening, be it opportunistic screening or an organized program, have observed very different effects on cervical cancer incidence and mortality [4]. Cervical cancer screening programs are in place in all of the Nordic countries, and the existence of population-based cancer registries, which have been monitoring the incidence and prevalence of all cancers since the 1950s, provide the opportunity to observe the effect of cervical cancer screening in each Nordic country (Figure 1) [5].

Pap smear was integrated into clinical practice in the Nordic countries in the 1960s. At that time, the age-standardized incidence rate of cervical cancer was about 15/10^5 ^in Norway and Finland. Subsequently, these two otherwise similar countries implemented profoundly different approaches regarding cervical cancer prevention. Finland introduced an organized screening program at the beginning of the 1960s. By the 1990s, cervical cancer incidence in Finland had been reduced to less than 4/10^5^, compared to 13/10^5 ^in Norway. Indeed, the organized screening program in Norway wasn't implemented until 1995 and cervical cancer incidence has since been reduced to 9/10^5^. In neighboring Denmark, the organized cervical cancer screening program was also introduced in the 1960s, when cervical cancer incidence was 31/10^5^, twice as high as in the other Nordic countries. By 2006, cervical cancer incidence in Denmark had dropped to 11/10^5^. These, findings indicate that both the incidence of and mortality from cervical cancer have declined remarkably in the Nordic countries following the introduction of organized screening.

However, in 2006 Denmark also reported 3.7 times more cervical cancer deaths (2.4/10^5^) than did Iceland (0.7/10^5^), and Finland had a cervical cancer incidence rate of 3.9/10^5^, which was 2.6 times lower than Denmark (10.3/10^5^). Clearly the Nordic countries, despite similar cultural and political backgrounds, and the existence of publicly funded healthcare systems, have achieved different levels of success in the prevention of cervical cancer.

The success of any cervical cancer prevention program relies on the interplay between the underlying risk factors that determine the spread of human papillomavirus infection in general population and the type of screening program. An organized program is more effective than an opportunistic program, and most importantly, high population coverage is a necessary factor for success. Furthermore, the existence and quality of the field and laboratory facilities for screening and diagnostic follow-up, as well as the facilities available for treating diagnosed lesions, are key elements of any screening program. Monitoring the patient path or "chain of action" for each patient with an abnormal screening result is of crucial importance. Only careful monitoring of these steps allows for the identification of potential shortcomings in the screening program, as well as areas for improvement [6].

Cervical cancer screening saves lives. Without screening, the rate of cervical cancer in the Nordic countries would most likely be higher than it was in the 1960s due to increased exposure to HPV infection. In Norway, despite the availability of an affordable and well-functioning health care system, as well as clearly defined recommendations for screening, follow-up, and treatment, cervical cancer is still a relevant health problem. Indeed, even after 30 years of opportunistic and 15 years of organized screening, some 300 cervical cancer cases are still diagnosed in the country every year.

There may be several explanations why organized programs in different countries do not reduce the burden of cervical cancer to the same degree. The extent of screening coverage is generally recognized as one of the most essential components for success, and coverage is improved by sending personal invitations to women at risk. In Norway, about 50% of cervical cancer cases arise from the 20% of the population who do not attend screening. The reason for non-attendance varies and deserves more research, but communication between health care providers and the target population seems to play an important role. Adequate communication of the importance of regular screening and the meaning of test results motivates women to attend subsequent screening rounds [7]. Communication is a key component of the doctor-patient relationship and it is likely that medical doctors play an integral role in improving patient knowledge and awareness. This is particularly true in Norway, where the screener is usually a family doctor or gynecologist, not a specialized midwife, as in Finland or Sweden. Educating and motivating the target population to attend screening is often given low priority by family doctors/gynecologists due to time constraints. It is also possible that a medical providers do not offer appropriate explanation, or are not sufficiently receptive to a woman's questions, thereby limiting communication between doctors and women who are to be screened. One remedy to improve education and motivation for screening in a Norwegian context is to increase the use of social media, which can be employed to market healthy behavior and disseminate health-related information.

However, it is not only the target screening population that needs to be educated. A study by the screening registry in Norway investigated clinical compliance with follow-up recommendations by observing how women with cytological abnormalities were followed-up in real life situations and comparing it with the recommendations. The observed adherence to recommendations regarding diagnostic procedures and treatment was good, provided that a screening smear indicated the presence of a high-grade lesion. The recommendations for the follow-up of low-grade or borderline lesions were often ignored, both in terms of timing and the type of follow-up test [8]. Such noncompliance results in sub-optimal performance of the screening regimen and can reduce the overall effectiveness of the program. Hence it is the extent to which recommended guidelines are followed in real life that determines the efficacy of the program, not the mere presence of formal recommendations.

The type of screening test used also plays an important role in cervical cancer screening. In this issue, Shi and colleagues compared the cost-effectiveness of three screening tests, HPV DNA testing and two different visual inspection methods. The authors concluded that *care*HPV in regular screening was more cost-effective than the two visual inspection methods. This finding is very encouraging, since it demonstrates that an objective and reliable primary screening test, with an acceptable performance and price, is now available for use in low-income countries. As is widely known, the conventional Pap smear screening method is not the ideal alternative in many low-resource settings, because the infrastructure for the test is neither available nor affordable.

The use of HPV DNA testing in screening has several potential advantages compared to Pap smear. First, HPV DNA testing has a higher sensitivity to detect underlying preinvasive lesions, as shown by two recently published randomized controlled trials [9,10]. It can also detect *in situ *adenocarcinomas, which often go undetected by Pap smear. Of equal importance, this in turn means that there is a low probability that HPV-negative women harbor preinvasive lesions or cervical cancer, resulting in a longer lead time compared to Pap smear. Consequently, it is possible to increase the screening interval, thus reducing the lifetime number of screening episodes and presumably improving both screening attendance and recommendation adherence. The reproducibility of HPV DNA testing is better than that of Pap smear, and the absence of borderline diagnoses simplifies the follow-up after a positive test. However, the high prevalence of high-risk HPV infections among women under 30 years of age suggests that an age-stratified implementation of primary screening by HPV DNA testing would be most beneficial, which complicates the overall recommendations. Also, communicating a positive HPV DNA test result to women can be a challenging and time consuming task for the doctor. Sexually transmitted oncogenic infection is a controversial topic, and cultural and religious beliefs undoubtedly influence an individual's reaction.

Cervical cancer screening has been compared to a chain that is only as strong as its weakest link. However, it is often very difficult to identify the weakest link. Careful auditing of the screening program and monitoring of the key indicators can help to define caveats in the screening program, which may be country-specific. Cervical cancer screening performance in the Nordic countries suggests that the availability of the screening test and a follow-up regimen alone do not guarantee a successful program. In addition to high coverage, continuous quality assurance of performance, both of the screening test and the follow-up regimen, is required for the program to fulfill its expectations.

The natural history of cervical cancer has been extensively studied and it is generally accepted that the disease progresses through preinvasive lesions following infection with sexually transmitted high-risk HPV types. This progression can be interrupted by screening. Mathematical models mimicking this natural history are applied in different populations as long as the key variables, as specified by the model, are available. For example, results from empirical studies, such as the age-specific high-risk HPV prevalence or data on sexual behavior in a given population are used to inform mathematical models estimating the natural history of cervical cancer. Empirical data on screening performance, however, can only be obtained by monitoring this performance within a given country. Hence, mathematical models, regardless of their level of sophistication, cannot be adequately calibrated without the use of country-specific screening data. For instance, the effect of HPV vaccination has been evaluated using mathematical models and the results support the notion that the vaccines will reduce the cervical cancer burden. These results reassured the policy makers who chose to allocate resources for vaccination. However, these results must be confirmed by surveillance studies utilizing population-based cancer registries, or by empirical studies.

## Conclusion

Modern health care systems are expected to generate the highest possible overall level of population health within a given set of resource constraints. Political and ethical considerations can further influence health care priorities. In this complex situation, findings from cost-effectiveness analyses will continue to impact and inform decision-making regarding health interventions. It is very important to emphasize, however, that cost-effectiveness analyses alone cannot guarantee the success of any program. Therefore, in the case of cervical cancer, it could be argued that the policy makers responsible for allocating resources for cervical cancer prevention have a duty to allocate resources *both *for cancer surveillance and screening program surveillance. This is particularly important for low-resource countries.

## Competing interests

The author declares that they have no competing interests.

## Pre-publication history

The pre-publication history for this paper can be accessed here:

http://www.biomedcentral.com/1471-2407/11/240/prepub
